# E-Cigarettes Use Behaviors in Japan: An Online Survey

**DOI:** 10.3390/ijerph19020892

**Published:** 2022-01-14

**Authors:** Shihoko Koyama, Takahiro Tabuchi, Isao Miyashiro

**Affiliations:** Cancer Control Center, Osaka International Cancer Institute, Osaka 541-8567, Japan; miyashir@biken.osaka-u.ac.jp

**Keywords:** e-cigarette, nicotine, tobacco, vaping

## Abstract

Electronic cigarette (e-cigarette) use has become increasingly widespread throughout the world, including in Japan. However, little is known about how e-cigarettes are used in Japan, a country with heavy restrictions on nicotine-containing e-liquids and/or vaping products. This study examined e-cigarette use (e-cigarette use duration, frequency of use, device type, electrical resistance, nicotine use, favorite e-liquid flavors) among users in Japan, through an online survey using a web-based self-reported questionnaire which included questions about sex, age, combustible cigarette and heated tobacco product (HTP) use behaviors. Of 4689 e-cigarettes users analyzed, 93.5% were men and 52.9% had been using e-cigarettes for 1–3 years. Over 80% used e-cigarettes every day; 62.3% used nicotine liquid, and half of the nicotine liquid users used nicotine salt. The most popular liquid flavor was fruit (prevalence: 68.1%), followed by tobacco (prevalence: 48.4%). While 50.9% were e-cigarette single users, 35.2% were dual users (e-cigarettes and cigarettes or HTPs) and 13.8% were triple user (e-cigarettes, cigarettes and HTPs). This is the first comprehensive survey of Japanese e-cigarette users and our finding suggest more than half use nicotine liquid, although e-cigarettes containing nicotine liquid have been prohibited by the Pharmaceutical Affairs Act since 2010 in Japan. The study also showed 49.1% of participants used cigarettes and/or HTPs concurrently (dual or triple users).

## 1. Introduction

Electronic cigarette (e-cigarette) use is a relatively new behavior that has spread rapidly worldwide. E-cigarettes are battery-operated devices typically designed to deliver nicotine and other additives to users in an aerosol form. The number and variety of e-cigarette brands is large and has been increasing [1,2]. This creates major challenges for e-cigarette-related research, as (1). there are no standardized assessments; (2). there are more than 2800 different e-cigarette devices from over 450 identified brands; (3). there are over 7700 unique e-liquid flavors [1]. Recently produced devices include a category commonly known as “sub-ohm”, which refers to devices with heating coils whose electrical resistance is well below 0.5 ohm [3]. The vaping process (e-cigarette use) also differs significantly from traditional smoking and varies among e-cigarette users (vapers). Sub-ohm devices produce large, exhaled vapor clouds, potentially leading to exposure to elevated levels of carbonyls, and induced detrimental effects on lung cells [3,4].

In Japan, e-cigarettes containing nicotine were evaluated by the Ministry of Health, Labour and Welfare, and the sale of these e-cigarettes has been prohibited by the Pharmaceutical Affairs Law since 2010. About 5% of young adults aged 15–29 years used e-cigarettes (with or without nicotine) in 2018. Although e-cigarette users are not popular at present, the flavors and design of e-cigarette products may attract young people and gain popularity in the youth community [5]. However, no study has investigated e-cigarette use behavior in detail, including dual and triple use with combustible cigarettes and heated tobacco products (HTPs) in Japan. Therefore, this study comprehensively examined e-cigarette use, including e-cigarette use duration, frequency of use, device type, electrical resistance, nicotine use, favorite e-liquid flavors and concurrent use with cigarettes and/or HTPs in Japan.

## 2. Materials and Methods

### 2.1. Study Population

Between 31 January and 31 May 2020, we conducted an online survey of people living in Japan who had used e-cigarette at least ≥1 time. It was administered using an online survey website (Google forms). 

We contacted known e-cigarette retailers and asked them to do the following: display an advertisement to recruit e-cigarette users to the study, alert potential participants about the study, and give us contact details of “e-cigarette user groups”. Twitter [6], Japanese e-cigarette user Facebook pages [7], and YouTube [8] sites where e-cigarette users posted messages were utilized to advertise the study. Similar methods have proved successful in other countries with a low prevalence of e-cigarette usage [9,10]. 

Participant information was shown online; after reading the information, potential participants confirmed their willingness to participate in the study by pressing a consent button. They were then able to proceed to the survey. Participation in the study was voluntary and no inducements to participate were offered. As possessing and/or using nicotine in e-cigarettes without a prescription is illegal in Japan, we anonymized the survey responses and did not collect identifying data (e.g., name, email, IP address) to allay any potential concerns about divulging illegal activity.

### 2.2. Measures

The online questionnaire was designed to collect participants’ demographics (sex, age), e-cigarette use behavior (e-cigarette use duration, frequency of e-cigarette use, the type of device, electrical resistance, nicotine use, favorite e-liquid flavors), and other tobacco (cigarettes and HTPs) use behaviors. Detailed wording was moderated using feedback from knowledgeable e-cigarette users (personal communication) before conducting the survey. 

E-cigarette use duration was determined by asking: “For how many years have you used e-cigarettes?” Five response options were possible: 1/2 year or less, 1/2–<1 year, 1–3 years, 4–<5 years, and 5 years or more. Frequency of e-cigarette use was determined by asking: “How often do you use e-cigarettes?”. Five response options were possible: less than once a month, twice or three times a month, once a week, twice or three times a week, and almost every day. Type of e-cigarette used was determined by asking: “Which of the following type of devices (Mod; Modification) do you use most often?” Four response options were possible: pod-system, regulated mod, mechanical mod, and unknown. Electrical resistance was determined by asking: “What is the electrical resistances of the atomizer of your most used devices?” Eight response options were possible: less than 0.09 ohm (Ω), 0.1~0.19 ohm, 0.2~0.29 ohm, 0.3~0.49 ohm, 0.5~0.8 ohm, 0.8~0.9 ohm, 1.0~1.2 ohm, and 1.2 ohm and more. 0.5 ohm or less of electrical resistance was defined as sub-ohm [3]. Nicotine use was determined by asking: “Have you ever use nicotine liquid?” Three response options were possible: no, yes (free-base nicotine), or yes (nicotine salt). Favorite e-liquid flavors were determined by asking: “Which e-liquid flavor do you like best?” Nine response options were possible: fruit (orange, lemon, melon etc.), dessert (chocolate, vanilla, cream etc.), drink (coffee, cola, energy drink etc.), refreshing (mint, herb etc.), menthol, tobacco, cannabidiol, tetrahydrocannabinol, and other flavor or prefer not to answer. 

Cigarette use status and HTP use status were determined by asking: “Have you ever smoked cigarettes? (used HTPs?)”. Five response options were possible “never”, “I tried them but it did not become a habit”, “used them habitually but have stopped now”, “often use” and “almost every day”. We combined these answers to form three categories according to the previous studies [11,12]; “never (never/I tried them but it did not become a habit)”, “former (used them habitually but have stopped now)”, and “current (often use/almost every day)”.

### 2.3. Ethical Consideration

The study was approved by the Institutional Review Board of Osaka International Cancer Institute (approval number: 19179) before initiation. The data were anonymized before use.

## 3. Results

Of 4769 people who accessed the online survey, 11 did not agree consent to participate, and 69 did not fit the eligibility criteria (not living in Japan or had not tried e-cigarettes). This left 4689 eligible participants in the final analysis.

### 3.1. Demographic Characteristics

Table 1 shows the demographic characteristics of the study subjects; 4384 (93.5%) were men; 33.6% and 33.2% of participants were 30–39 years old and 40–49 years old, respectively.

### 3.2. E-Cigarette Use Behaviors

Table 2 shows detailed e-cigarette use behaviors. About half (52.9%) of the participants had been using e-cigarettes for 1–3 years. Over 80% of participants used e-cigarettes almost every day; 62.3% of participants used the regulated Mod type of vape; 36.4% used 0.5 ohm or less of electrical resistance (sub-ohm); 62.3% of participants used nicotine liquid e-cigarettes, and half of nicotine liquid users used nicotine salt.

### 3.3. Favorite E-Cigarette Flavors

**Figure 1:**
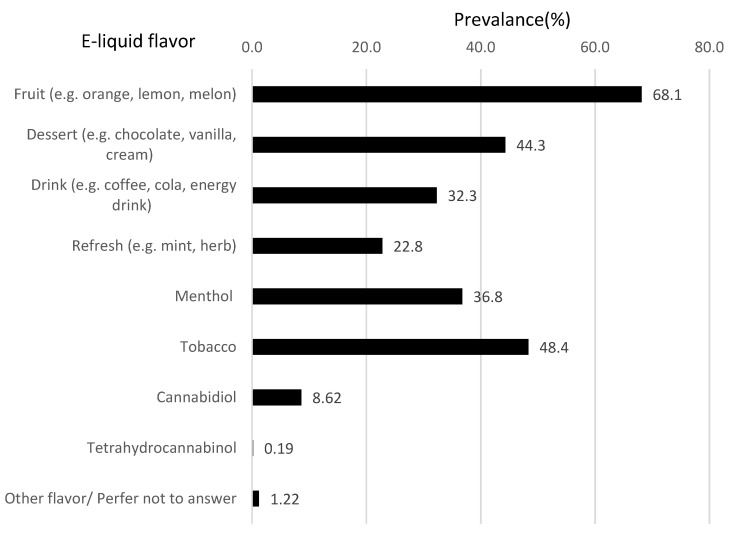
Favorite e-liquid flavors (multiple choice) among Japanese e-cigarette users.

Figure 1 shows the percentages of favorite e-cigarette flavors (multiple answers possible). Fruit was the most popular flavor (prevalence: 68.1%) followed by tobacco (prevalence: 48.4%).

### 3.4. Other Tobacco Use Behaviors (Cigarette and HTPs)

Table 3 shows other tobacco use behaviors (cigarette and HTPs) according to e-cigarette use duration: 50.9% of e-cigarette users used only e-cigarettes (single user); 35.2% were dual users (e-cigarettes and cigarettes (18.9%) or HTPs (16.3%)) and 13.8% were triple users (e-cigarettes, cigarettes and HTPs). Of short-term e-cigarette users (less than 1/2 year), 43.5% were dual or triple users. Of long-term e-cigarette users (5 years or more) 55.2% were dual or triple users.

## 4. Discussion

The current research objective was novel in Japan, especially the detailed consideration of e-cigarette use behaviors. Of e-cigarette users, 62.3% were nicotine liquid users and 37.7% were non-nicotine liquid users. Our estimates of nicotine liquid use are lower than those reported in two previous studies [13,14] for adult e-cigarette users in the U.S., which found 69.2% and 89.2%, respectively, usually used nicotine-containing e-cigarettes. This is probably because sales of nicotine-containing e-cigarettes are allowed in the U.S., while e-cigarettes containing nicotine liquid have been prohibited by the Pharmaceutical Affairs Act since 2010 in Japan. However, due to the ease of personal importation through the internet, a relatively high number of e-cigarette users used nicotine liquid in Japan. While less than 50% of newer e-cigarette users (using for less than 1 year) used nicotine-liquid, more than 50% of longer-term users (using for more than 1 year) used nicotine-liquid. (Supplementary Table S1).

Additionally, 28.9% of e-cigarette users used nicotine salt, suggesting these users may have higher risk of exposure to high-level nicotine and thus could easily become addicted to e-cigarettes. Nicotine salt is synthetically generated by alkaline free-base nicotine and any acid (lactic, benzoic, levulinic) [15,16]. Compared with alkaline free-base nicotine, nicotine salt reduces both harshness and bitterness, and increases sweetness and smoothness for e-cigarette users [17]; it is also more likely to alter lung epithelium inflammatory responses, which could increase the risk of respiratory illness [18].

Furthermore, the current study was also the first to report that the prevalence of sub-ohm users was 36.4% in Japan, suggesting another health risk among e-cigarette users due to exposure to elevated levels of carbonyls, and induced detrimental effects on lung cells [3,4]. Previous studies showed that younger, and mostly male, users were attracted to the high-power vaping style (nearly equivalent to ‘sub-ohming’) using more powerful devices and direct lung inhalation [19,20], suggesting the necessity of monitoring young and male users in future. 

Among e-cigarette flavors, fruit flavors were the most popular, followed by tobacco which is consistent with previous studies from the U.S. and Canada [21,22,23]. As previous research has shown that an interest in the fruit flavor was the main reason for trying e-cigarettes in Japan [5], we need to monitor e-cigarette flavors as well as nicotine salt and sub-ohming. In Japan, use of cannabidiol is legal, but use of tetrahydrocannabinol (i.e., ingredients designated for narcotics) is illegal. In this survey the proportion of cannabidiol use was 8.62%, while that of tetrahydrocannabidiol use was 0.19%.

Among e-cigarette users, 35.2% were dual users, and 13.8% were triple users. Nearly 50% of both long-term e-cigarette users (5 years or more) and short-term users (1/2 years or less), used tobacco products (dual or triple users). A previous study in New Zealand showed that newer e-cigarette users (under 1 year) were more likely to use cigarettes concurrently (30.2%) compared with long term e-cigarette users (2 years or more: 10.4%) [9]. Further, a previous study among adolescents in Ireland showed that 64.6% of e-cigarette users concurrently use cigarettes (dual users) [24]. In the present study, 49.1% of e-cigarette users used tobacco products concurrently, suggesting that these users have a higher risk of health deterioration.

The present study has several limitations. First, the participants were not randomly selected; rather, they were recruited through e-cigarette user/retailer networks. This may have led to a selection bias. Compared with previous studies in other countries, fewer youth e-cigarette users were included in this study. Because information on the prevalence of e-cigarette (not HTP) use in Japan was not available in the national survey, we were unable to determine whether e-cigarette users in the present study represent the overall Japanese e-cigarette user population. This limited the generalizability of the study. Second, this study was a simple descriptive analysis. Smoking variables were self-reported without biomarker validation, but the reliability of self-reported smoking behavior was generally thought to be high [25]. However, no previous study has investigated Japanese e-cigarette users and our study included over 4000 participants. As patterns of e-cigarette usage are complicated, understanding the current situation of e-cigarette users, as revealed in our study, may be useful for public health. 

## 5. Conclusions

In conclusion, this is the first comprehensive survey of Japanese e-cigarette users, suggesting more than half use nicotine liquid, despite e-cigarettes with nicotine liquid having been prohibited by the Pharmaceutical Affairs Act since 2010 in Japan. Nearly half, 49.1%, of e-cigarette users used cigarettes and/or HTPs concurrently (dual or triple users).

## Figures and Tables

**Table 1:** Demographic characteristics of study subjects (*n* = 4689).

	Number	%
Sex		
Men	4384	93.5
Women	281	5.99
Other	24	0.51
Age		
16–19	6	0.13
20–24	328	7.0
25–29	543	11.6
30–34	752	16.0
35–39	825	17.6
40–44	869	18.5
45–49	689	14.7
50–54	406	8.66
55–59	201	4.29
60–71	70	1.5

**Table 2:** Detailed e-cigarette use behaviors (*n* = 4689).

	Number	%
E-cigarette use duration		
1/2 year or less	223	4.76
1/2–<1 year	434	9.26
1–3 years	2479	52.9
4–<5 years	914	19.5
5 years or more	639	13.6
Frequency of e-cigarette use		
Once a month	84	1.79
Twice or three times a month	74	1.58
Once a week	71	1.51
Twice or three times a week	412	8.79
Almost everyday	4048	86.3
Type of e-cigarette		
Pod-system	881	18.8
Regulated Mod	2922	62.3
Mechanical Mod	821	17.5
Unknown	65	1.39
Electrical resistance		
Less than 0.09 ohm (Ω) ^a^	48	1.0
0.1~0.19 ohm ^a^	366	7.8
0.2~0.29 ohm ^a^	443	9.4
0.3~0.49 ohm ^a^	848	18.1
0.5~0.8 ohm	1226	26.1
0.8~0.9 ohm	512	10.9
1.0~1.2 ohm	689	14.7
1.2 ohm and more	362	7.7
Unknown	195	4.2
Nicotine use		
No	1766	37.7
Yes (free-base nicotine) ^b^	1569	33.5
Yes (nicotine salt)	1354	28.9

^a^ Sub-ohm, ^b^ Free-base nicotine = alkaline free-base nicotine only.

**Table 3:** Use status of tobacco (cigarettes and heated tobacco products) according to e-cigarette use duration (*n* = 4689).

Use Status			Total	E-Cigarette Use Duration				
Cigarettes	HTPs ^a^			1/2 Year or Less	1/2–<1 Year	1–3 Years	4–<5 Years	5 Years or More
			*n* (col %)	*n* (col %)	*n* (col %)	*n* (col %)	*n* (col %)	*n* (col %)
never	never		422 (9.0)	43 (19.3)	70 (16.1)	233 (9.4)	48 (5.3)	28 (4.4)
never	former		6 (0.1)	0 (0.0)	3 (0.7)	2 (0.1)	0 (0.0)	1 (0.2)
former	never		1125 (24.0)	46 (20.6)	67 (15.4)	544 (21.9)	272 (29.8)	196 (30.7)
former	former		836 (17.8)	37 (16.6)	87 (20.0)	512 (20.7)	139 (15.2)	61 (9.5)
single user total			2389 (50.9)	126 (56.5)	227 (52.3)	1291 (52.1)	459 (50.2)	286 (44.8)
never	current	(dual user)	42 (0.9)	7 (3.1)	7 (1.6)	22 (0.9)	4 (0.4)	2 (0.3)
current	never	(dual user)	633 (13.5)	33 (14.8)	44 (10.1)	316 (12.7)	142 (15.5)	98 (15.3)
former	current	(dual user)	722 (15.4)	24 (10.8)	65 (15.0)	376 (15.2)	154 (16.8)	103 (16.1)
current	former	(dual user)	254 (5.4)	11 (4.9)	28 (6.5)	146 (5.9)	36 (3.9)	33 (5.2)
dual user total			1651 (35.2)	75 (33.6)	144 (33.2)	860 (34.7)	336 (36.8)	236 (36.9)
current	current	(triple user)	649 (13.8)	22 (9.9)	63 (14.5)	328 (13.2)	119 (13.0)	117 (18.3)
dual & triple user total			2300 (49.1)	97 (43.5)	207 (47.7)	1188 (47.9)	455 (49.8)	353 (55.2)
Total			4689 (100.0)	223 (100.0)	434 (100.0)	2479 (100.0)	914 (100.0)	639 (100.0)

^a^ HTP; heated tobacco products.

## Data Availability

The data presented in this study are available on request from the corresponding author.

## Supplementary Materials

The following are available online at https://www.mdpi.com/article/10.3390/ijerph19020892/s1, Table S1: Nicotine liquid use according to e-cigarette use duration (*n* = 4689).
