# Supplementary material for: E-Cigarettes Use Behaviors in Japan: An Online Survey
Source: Int J Environ Res Public Health. 2022 Jan 14;19(2):892. doi: 10.3390/ijerph19020892 (PMC8775432; doi:10.3390/ijerph19020892)
Supplement: Supplementary file 1 [file ijerph-19-00892-s001.zip › ijerph-1516137-supplementary.pdf]

Supplementary file

Table S1. Nicotine liquid use according to e-cigarette use duration (n = 4,689).

	E-cigarette use duration											
	1/2 years or less		1/2-1 years		1-3 years		4-5 years		5 years or more		Total	
Nicotine use	n	(col %)	n	(col %)	n	(col %)	n	(col %)	n	(col %)	n	(col %)
No	156	(70.0)	259	(59.7)	968	(39.0)	251	(27.5)	132	(20.7)	1766	(37.7)
Yes (free-base nicotine)	38	(17.0)	80	(18.4)	814	(32.8)	362	(39.6)	275	(43.0)	1569	(33.5)
Yes (nicotine salt)	29	(13.0)	95	(21.9)	697	(28.1)	301	(32.9)	232	(36.3)	1354	(28.9)
Total	223	(100.0)	434	(100.0)	2479	(100.0)	914	(100.0)	639	(100.0)	4689	(100.0)
